# Estimating tissue-specific peptide abundance from public RNA-Seq data

**DOI:** 10.3389/fgene.2023.1082168

**Published:** 2023-01-12

**Authors:** Angela Frentzen, Jason A. Greenbaum, Haeuk Kim, Bjoern Peters, Zeynep Koşaloğlu-Yalçın

**Affiliations:** ^1^ Center for Infectious Disease and Vaccine Research, La Jolla Institute for Immunology, San Diego, CA, United States; ^2^ Department of Medicine, University of California, San Diego, San Diego, CA, United States

**Keywords:** RNA-Seq, RNA sequencing, peptide (pep), prediction, ligands, tool, cancer

## Abstract

Several novel MHC class I epitope prediction tools additionally incorporate the abundance levels of the peptides’ source antigens and have shown improved performance for predicting immunogenicity. Such tools require the user to input the MHC alleles and peptide sequences of interest, as well as the abundance levels of the peptides’ source proteins. However, such expression data is often not directly available to users, and retrieving the expression level of a peptide’s source antigen from public databases is not trivial. We have developed the Peptide eXpression annotator (pepX), which takes a peptide as input, identifies from which proteins the peptide can be derived, and returns an estimate of the expression level of those source proteins from selected public databases. We have also investigated how the abundance level of a peptide can be best estimated in cases when it can originate from multiple transcripts and proteins and found that summing up transcript-level expression values performs best in distinguishing ligands from decoy peptides.

## Introduction

Presentation of peptides on the cell surface by major histocompatibility complex (MHC) class I molecules is crucial for CD8^+^ T cell-mediated immune responses, including those against viral infections and tumors. Receptors on the surface of T cells (TCRs) scan MHC-bound peptides, and peptides that lead to T Cell activation and proliferation are referred to as T Cell epitopes. Numerous computational tools have been developed to predict which peptides will bind to MHC molecules and likely be recognized as epitopes (Peters et al., 2020). Several novel MHC class I epitope prediction tools additionally incorporate the abundance levels of the peptides’ source antigens and have shown improved performance (Abelin et al., 2017; Sarkizova et al., 2020; Garcia Alvarez et al., 2022; Kosaloglu-Yalcin et al., 2022) for predicting immunogenicity. Such tools require the user to input the MHC alleles and peptide sequences of interest, as well as the abundance levels of the peptides’ source proteins. However, such expression data is often not directly available to users, and retrieving the expression level of a peptide’s source antigen from public databases is not trivial. First, it needs to be determined from which protein(s) the peptide of interest can be derived. Then, the expression values of those proteins need to be fetched from public expression datasets, and data have to be aggregated to account for variability between different individuals and the availability of the same peptide from multiple transcript variants and/or multiple genes.

To address these issues, we have developed the Peptide eXpression annotator (pepX), which takes a peptide as input, identifies from which proteins the peptide can be derived, and returns an estimate of the expression level of those source proteins. In this study, we have also investigated how the abundance level of a peptide can be best estimated in cases when it can originate from multiple source antigens. RNA-Seq gene and transcript expression quantification can be calculated, for example, as FPKM (fragments per kilobase of transcript per million fragments mapped), RPKM (reads per kilobase of exon per million reads mapped), or TPM (transcripts per million). In this study, we chose to use TPM to quantify gene expression. TPM values can be calculated on the transcript level by counting the RNA-Seq reads covering each transcript sequence. TPM values can also be calculated on the gene level by counting RNA-Seq reads covering each transcript encoded by a gene. Here, we provide insights into the differences between using gene-level and transcript-level TPM values for estimating peptide abundances.

We utilize expression data from several public databases, including The Cancer Genome Atlas (TCGA) (Cancer Genome Atlas Research et al., 2013), Genotype-Tissue Expression (GTEx) (Carithers and Moore, 2015), Cancer Cell Line Encyclopedia (CCLE) (Ghandi et al., 2019), and the Human Protein Atlas (HPA) (Uhlen et al., 2010). pepX is freely available as a web-based resource at http://tools.iedb.org/pepx.

## Materials and methods

### RNA-Seq datasets

The Riaz and Hugo bulk RNA datasets used in this study are available under BioProject accession numbers PRJNA356761 (Riaz et al., 2017) and PRJNA312948 (Hugo et al., 2016), respectively. Raw RNA-Seq reads were downloaded and processed using an in-house RNA-Seq mapping and analysis pipeline to calculate gene-level TPM values.

### Expression datasets

Pre-calculated gene-level and transcript-level TPM values for the TCGA Pan-cancer cohort for 33 cancer types were downloaded from the UCSC Xena data pages (Goldman et al., 2020).

Pre-calculated gene-level and transcript-level TPM values for 256 healthy tissues were downloaded from the Human Protein Atlas (HPA) (Uhlen et al., 2010).

Pre-calculated gene-level and transcript-level TPM values for 54 healthy tissue subtypes were downloaded from The Genotype-Tissue Expression (GTEx) project data portal (Carithers and Moore, 2015). Median TPM values were calculated for each of the 31 main tissue types.

Pre-calculated gene-level and transcript-level TPM values for 1,019 cell lines were downloaded from the Cancer Cell Line Encyclopedia (CCLE) (Ghandi et al., 2019).

All datasets were downloaded in July 2022.

### MHC class I ligand elution datasets

The Trolle dataset consisted of 15,524 non-redundant HLA class I ligands eluted from mono-allelic HeLa cells transfected with five different HLA class I alleles (Trolle et al., 2016). This dataset was downloaded from the IEDB (Vita et al., 2019) under the accession number 1000685 (http://www.iedb.org/subID/1000685).

The Abelin dataset contained 22,310 non-redundant eluted ligands from mono-allelic B721.221 cells transfected with 16 different HLA class I alleles. The dataset was retrieved from the supplementary materials of the original publication (Abelin et al., 2017). Abelin et al. also provided matched RNA-Seq data for four replicates under BioProject accession number PRJNA360601. Raw RNA-Seq reads were downloaded and processed using an in-house RNA-Seq mapping and analysis pipeline to calculate gene-level TPM values. Median TPM values of the four replicates were used.

The HLA Ligand Atlas consisted of tissue-specific HLA ligands from 23 healthy tissue types (Marcu et al., 2021). The dataset contained 223,246 non-redundant peptides and 675,346 peptide tissue pairs. We downloaded the data from the HLA Ligand Atlas data pages (downloaded in September 2022).

The Shinkawa dataset contained 2,352 non-redundant HLA class I eluted ligands from a HCT15/β2 cell line. The dataset was retrieved from the supplementary materials of the original publication (Shinkawa et al., 2021).

The Pyke dataset contained 34,090 ligands eluted from mono-allelic K562 cell lines transfected with 25 different HLA alleles. The Pyke Cancer dataset contained 31,660 ligands eluted from 12 tissue samples of colorectal and lung cancer patients. Both datasets were retrieved from the Supplementary Material S1 of the original publication (Pyke et al., 2021).

The Sarkizova dataset contained 140,918 eluted ligands from B721.221 cells transfected with 79 different HLA class I alleles. The dataset was retrieved from the supplementary materials of the original publication (Sarkizova et al., 2020).

### PepX

The backend of pepX is a PostgretSQL database that is populated with all possible 8-15mers from the human proteome linked to TPM data from several publicly available databases.

**FIGURE 1:**
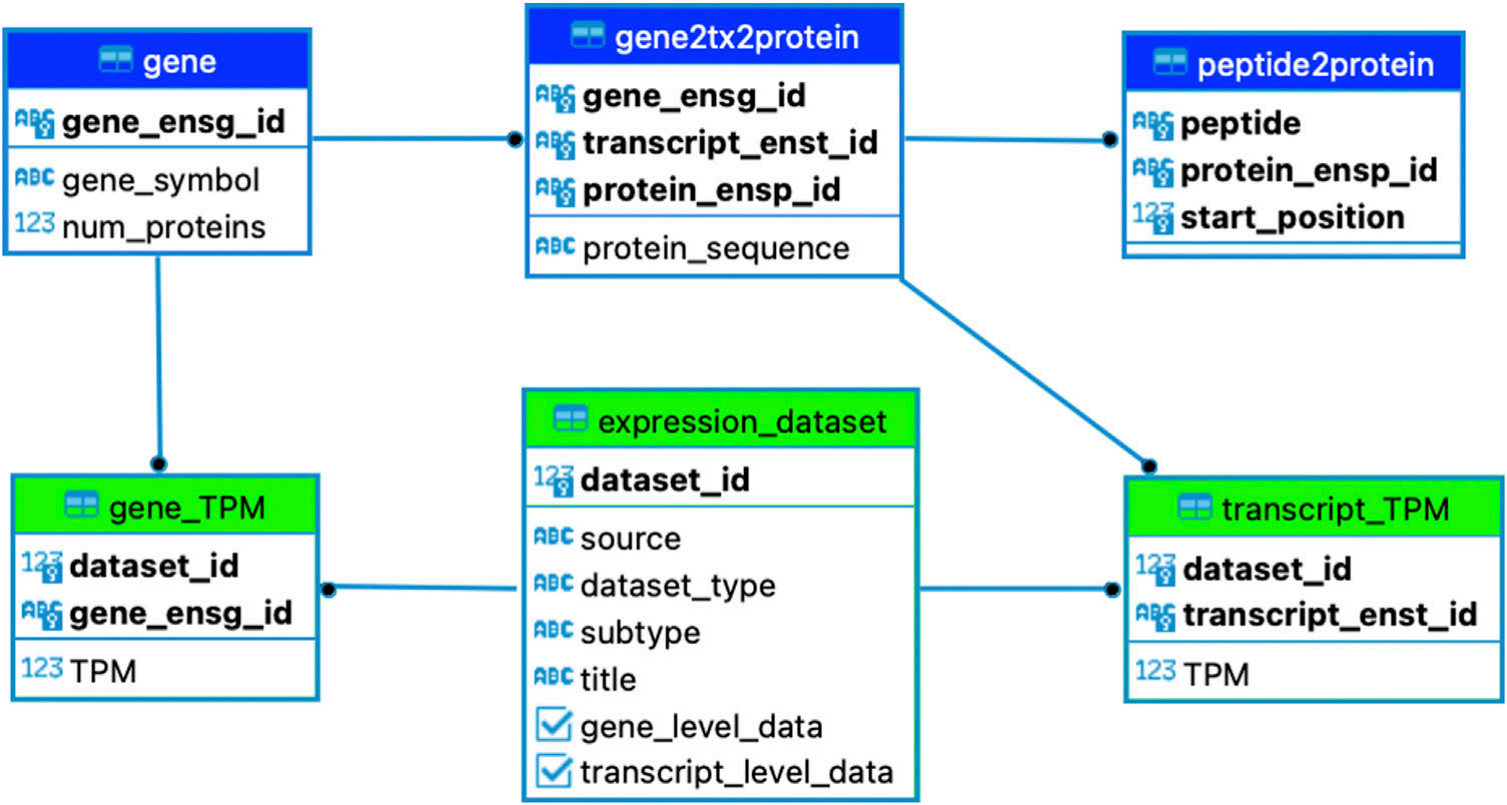
pepX Database Schema. The three proteome-related tables of the database (in blue) catalog peptides, proteins, transcripts and genes. The three expression-related tables of the database (in green) track gene- and transcript-level TPM data and associated study details.

A partial entity-relation diagram of the core tables in the pepX database is shown in Figure 1. The proteome tables of the database were populated by retrieving all possible 8-15mers from the GRCh38 Ensembl proteome (release-106). All peptides are linked to their associated protein sequences, as well as gene, transcript, and protein identifiers. The ‘gene’ table includes the unique Ensembl gene (ENSG) identifiers, their gene symbols, and the number of proteins encoded by the gene. The ‘gene2tx2protein’ table contains one row per protein/transcript and maps the Ensembl gene identifier to their corresponding protein (ENSP) and transcript (ENST) IDs. This table also includes the full protein sequence. The ‘peptide2protein’ table maps each 8-15mer to their corresponding Ensembl protein ids, keeping note of the zero-indexed start position of the kmer within the full protein sequence. This schema allows pepX to quickly lookup which genes and transcripts are linked to a given peptide.

We included expression data from six public databases in pepX, namely HPA, GTEx, TCGA, CCLE, and RNA-Seq data of a B721.221 cell line (Abelin et al., 2017) as well as a HeLa cell line (Cantarella et al., 2019). Each has several subtypes, which are cancer types in the case of TCGA (e.g. BRCA, COAD, PAAD), tissue-types in the case of HPA and GTEx (e.g. Skin, Stomach, Thyroid Gland), and cell-lines in the case of CCLE (e.g. HELA_CERVIX, HCC56_LARGE_INTESTINE). These subtypes and their external dataset source are listed in the ‘expression_dataset’ table, each with Boolean values to indicate if gene- and transcript-level data are available (Figure 1). All entries in the ‘expression_dataset’ table contain a unique ‘dataset_id’ that is used to map TPM values in the ‘gene_TPM’ and ‘transcript_TPM’ tables back to the associated dataset. The ‘gene_TPM’ table maps TPM’s from a given subtype to an Ensembl gene id, while the ‘transcript_TPM’ table maps TPMs to the Ensembl transcript id. This structure allows pepX to quickly grab all TPMs linked to a given gene or transcript for a given study.

Several views have been created that efficiently perform the joins necessary to provide peptide-level and gene/transcript-level TPM output, given a list of peptides and dataset ID as input. In addition to the raw TPMs, scaled TPMs are calculated for each of the source proteins as:

$${scaled}_{tpm}=TPM*\frac{\#\;of\;proteins\;for\;the\;gene\;in\;which\;the\;peptide\;is\;found}{\#of\;total\;proteins\;for\;the\;gene}$$

For each peptide, the total TPM, median TPM, and maximum TPM are also provided.

### Statistical analysis

R/Bioconductor was used for all statistical analyses. The following significance levels were used in all figures: ns: *p* > .05, *: p≤.05, **: p≤.01, ***: p≤.001, ****: p≤.0001. All statistical tests are paired Wilcoxon tests, unless otherwise indicated.

### RNA-Seq mapping pipeline

Reads mapping to tRNA, rRNA, adapter sequences, and spike-in controls were filtered with Bowtie 2 (v2.1.0). Remaining reads were mapped go the GRCh38 reference genome with Gencode v27 annotations using STAR (v2.6.1). Low complexity reads (DUST >4) were removed from the BAMs with PRINSEQ Lite (v0.20.3) before counting reads with FeatureCount (v1.6.5).

## Results

### Gene expression data from public databases correlate well with individual patient-derived RNA-Seq data

The TCGA program sequenced thousands of tumor and matched normal samples spanning 33 cancer types. For each cancer type, the number of patients analyzed varies, from 1,211 patients with breast cancer (TCGA-BRCA) to 45 patients with Cholangiocarcinoma (TCGA-CHOL, Supplementary Figure S1). This means when looking for the expression of a gene of interest in a specific cancer type, there are several expression values from different patients which need to be aggregated and summarized to derive one expression value per gene.

**FIGURE 2:**
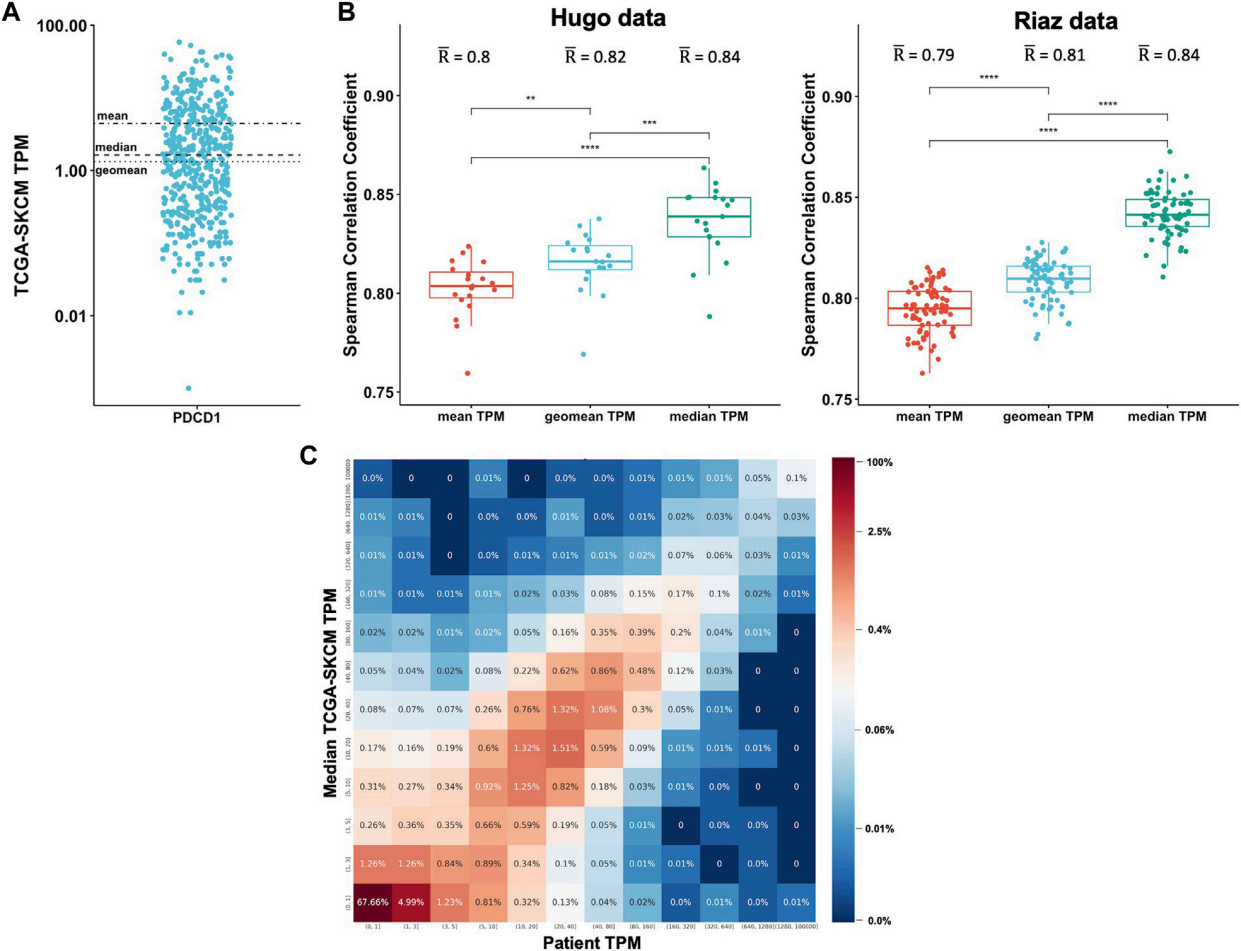
Expression data from TCGA correlates well with patient-specific RNA-Seq data. **(A)**. The mean, median, and geomean TPM were calculated for the gene PDCD1 for the 470 TCGA-SKCM patients (each dot represents one patient). **(B)**. For each patient in the Hugo (Hugo et al., 2016) and Riaz (Riaz et al., 2017) datasets, all genes were considered and TPM values were correlated to the TCGA-SKCM mean, median, and geomean TPM. Spearman correlations coefficients were calculated. **(C)**. For each patient, the TPM values were separated into ranges for both the patient-specific (*x*-axis) and the TCGA median (*y*-axis) TPM values. For each TPM range combination, the fraction of genes expressed within the corresponding TPM ranges is shown as a percentage and is also color-coded.

We sought to investigate what the best method is to aggregate TPM values from different patients and to determine how well the RNA-Seq data from TCGA correlates with patient-matched RNA-Seq. We obtained patient-matched RNA-Seq data from two melanoma studies published by Hugo et al. (Hugo et al., 2016) and Riaz et al. (Riaz et al., 2017) and compared the TPM values to the TCGA skin cancer samples (TCGA-SKCM). We aggregated the data over the 470 TCGA-SKCM patients by calculating the mean, median, and geometric mean TPM for each gene. Given the statistical background of how these metrics are calculated, it is expected that the values can significantly vary. The TPM values for PD-1 (PDCD1), for example, range between 0 and 60 in the TCGA-SKCM cohort (Figure 2A). The mean is 4.5, the median is 1.6, and the geometric mean is 1.3. For each patient in the Hugo and Riaz datasets, we considered all genes and calculated how well the TPMs correlate to the TCGA-SKCM mean, median, and geometric mean. We found that the values of the three metrics significantly vary from each other (Kruskal-Wallis test, *p* < .0001) and that the median TCGA-SKCM correlates best with patient-specific TPM values in both datasets (Wilcoxon test, *p* < .001, Figure 2B).

To determine the overall correlation between the patient-specific TPM values and the TCGA-SKCM median TPM values, we combined the Hugo and Riaz datasets and found a significant correlation (Spearman correlation coefficient r = .82). To get a better overview of the correlation, we separated the TPM values for each patient into ranges for both the patient-specific and the TCGA median TPM values to generate a 2-dimensional matrix. We then analyzed each TPM range combination and calculated the fraction of genes expressed within the corresponding TPM ranges (Figure 2C). We found that 67% of genes are expressed at low levels in both the Hugo and Riaz cohort and in the TCGA-SKCM cohort, with TPM values of <1. The majority of genes are expressed at similar levels in both cohorts, as demonstrated by an enrichment of genes on the diagonal in Figure 2C. We observed similar results when we analyzed a smaller set of in-house patients with six different cancer types (Supplementary Figure S2).

Taken together, these findings show that TPM values from a public database like TCGA are suitable for estimating gene expression in a patient sample if patient-specific RNA-Seq is not available.

### Retrieving peptide abundance from public databases and aggregating expression levels from different source antigens

We developed pepX, a tool for estimating a peptide’s expression level based upon the source antigen(s) in which it is contained. pepX takes a list of peptides and a public dataset identifier as input and returns the expression level of each protein the peptide was found in. pepX also provides aggregated expression levels for peptides that can be retrieved from multiple transcripts and proteins. The expression levels can be retrieved from a number of public databases, including The Cancer Genome Atlas (TCGA), CCLE (The Cancer Cell Line Encyclopedia) (Ghandi et al., 2019), HPA (The Human Protein Atlas) (Uhlen et al., 2010), and GTEx (The Genotype-Tissue Expression Project) (Carithers and Moore, 2015). These datasets provide expression values on the gene level as well as the transcript level. We used pepX to investigate different ways of estimating peptide abundance and the differences in using gene-level and transcript-level TPM values.

**FIGURE 3:**
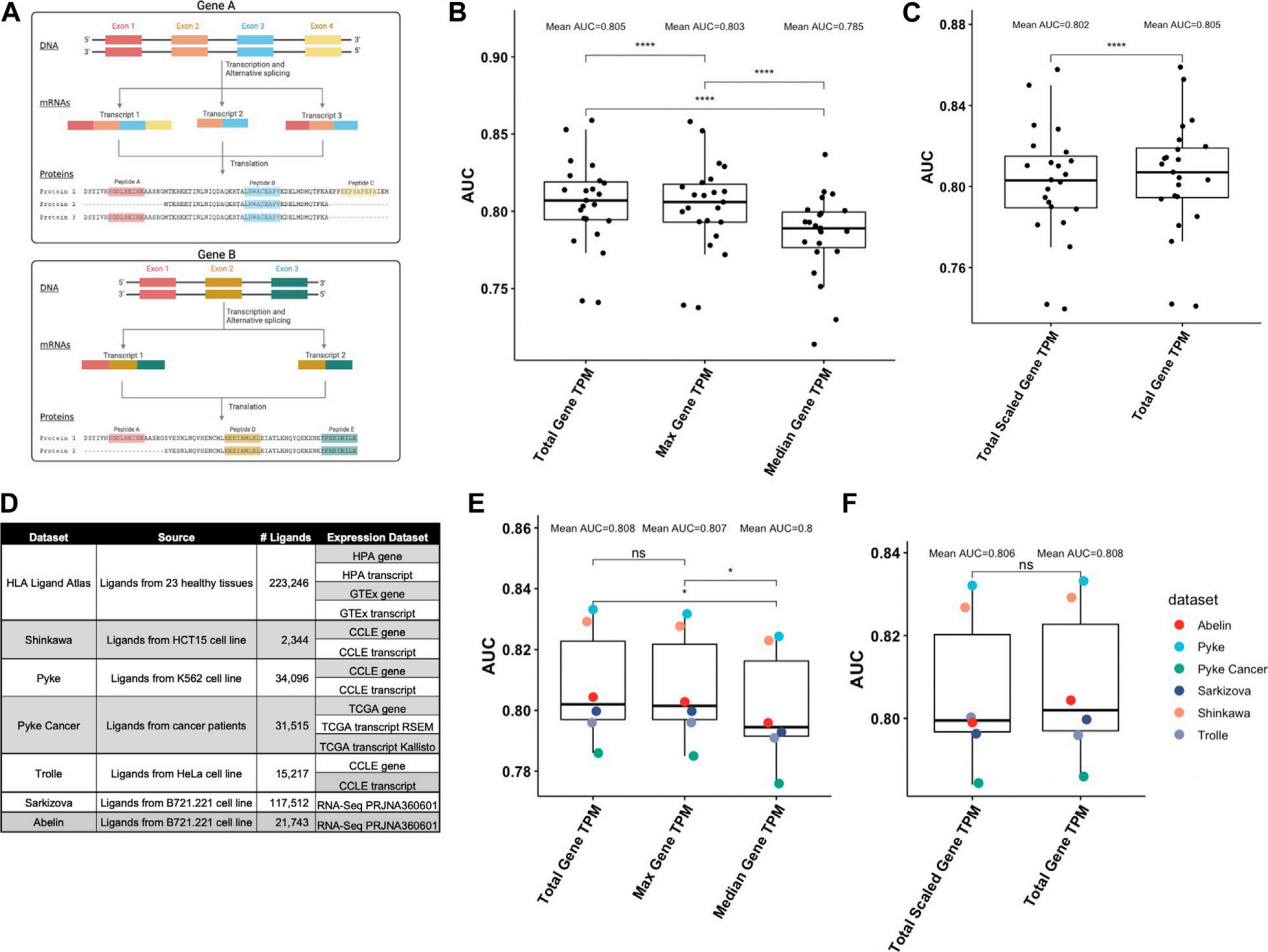
Considerations for retrieving peptide abundance levels. Due to alternative splicing, genes can produce multiple different proteins. **(A)** The different protein sequences usually share amino acid stretches encoded by the same exons. It is also possible, that different genes share amino acid stretches, particularly genes from the same gene family. Peptide A (highlighted in red) for example can be retrieved from two proteins of Gene A and from one protein of Gene B, while Peptide B (highlighted in blue) can be retrieved from three proteins of Gene. **(B)**. Performance comparison of different ways to aggregate TPM values of multiple source proteins in distinguishing ligands of the HLA Ligand Atlas from decoy peptides. Summing up TPM values (total TPM) from all genes a peptide can be retrieved from performs best, followed by using the maximum TPM of all genes (Wilcoxon Test, p≤.0001). **(C)**. Performance comparison of scaling the TPM values considering the number of proteins a gene encodes and the number of proteins a peptide occurs in for ligands from the HLA Ligand Atlas. The total TPM significantly outperformed the total scaled TPM values (Wilcoxon Test, p ≤ .0001). **(D)**. Ligand elution datasets used in this study and the expression datasets we used to retrieve abundance levels. **(E)**. Performance comparison of different ways to aggregate TPM values of multiple source proteins in distinguishing ligands of the six validation datasets from decoy peptides. **(F)**. Performance comparison of total TPM and total scaled TPM proteins in distinguishing ligands of the six validation datasets from decoy peptides.

As illustrated in Figure 3A, it is possible that the exact same peptide can be found in different proteins encoded by different genes (e.g., Peptide A in Figure 3A can be retrieved from two proteins of Gene A and from one protein of Gene B). To analyze the extent of this, we considered the set of unique peptides in the HLA Ligand Atlas (n = 223,246) (Marcu et al., 2021) and investigated the number of possible source proteins for each peptide. We found that 88% of peptides can be retrieved from protein sequences corresponding to exactly one Ensemble gene id. We investigated the remaining 12% of peptides that could be retrieved from different gene ids and found that, for the majority of cases (96%), the corresponding genes belonged to the same gene family. As gene families are formed by duplication of a single original gene, genes that are categorized into families usually share nucleotide and protein sequences. It is thus not surprising that a peptide can occur in multiple proteins that are encoded by genes that are part of a gene family. It is, however, not clear how the abundance of such peptides should be measured, as there are several options: 1) using the median TPM of all genes, 2) using the maximum TPM among all genes, or 3) summing up the TPM values of all genes.

### Summing up expression levels from different source antigens provides the most accurate estimation of peptide abundance

We used peptides from the HLA Ligand Atlas for validation and analysis of pepX performance. The HLA Ligand Atlas contains tissue-specific HLA ligands from 23 healthy tissue types (Marcu et al., 2021). The dataset consisted of 223,246 non-redundant peptides and 675,346 peptide tissue pairs. We wanted to investigate how the abundance level of these peptides could be best estimated using gene-level TPM values. We generated a set of decoy peptides by randomly selecting length-matched peptides from the human proteome and assessed the performance of different metrics in distinguishing true ligands from the set of random decoy peptides. The Human Protein Atlas was used as the expression dataset by matching the tissue types represented in the HLA Ligand Atlas **(** Supplementary Table S1 **)**.

We evaluated all three options for aggregating expression values across different source antigens: 1) using the median TPM of all genes, 2) using the maximum TPM among all genes, or 3) summing up the TPM values of all genes. We used pepX to identify from which proteins each peptide could be derived from and retrieved expression levels of those source proteins. We evaluated the performance of each TPM aggregation method in distinguishing peptides from the HLA Ligand Atlas from the set of decoy peptides. The Area under the Receiver Operating Characteristics (ROC) Curve (AUC) was used to measure performance. We found that the values of the three metrics significantly vary from each other (Kruskal-Wallis test, *p* = .01034) and that with a mean AUC of .805, summing up TPM values (total TPM) performs best, followed by using the maximum TPM among all genes (mean AUC = .803) and using the median TPM of all genes (mean AUC = .785, Wilcoxon test, *p* < .0001, Figure 3B).

Another detail we wanted to investigate was that each gene can be transcribed into different transcripts and thus translated into different proteins. The different transcripts correspond to different splice variants that are found in different tissue types, developmental stages, *etc.* However, the expression values of the different transcripts of a gene are collapsed into a single expression value when generating gene-level expression data. We hypothesized that, when using such gene-level TPM data, it might be important to consider in how many of a gene’s transcripts the peptide occurs (e.g., Peptide B in Figure 3A occurs in three different proteins from Gene A, while Peptide C occurs only in one). We developed a ‘scaled TPM’, which considers the number of proteins in which the peptide is found and the total number of proteins for the gene (detailed in the Methods section). However, with a mean AUC of .802, this scaled TPM did not improve the performance in predicting peptides from the HLA Ligand Atlas (Figure 3C).

To validate these findings, we gathered additional ligand elution datasets and matched them to their corresponding expression datasets (Figure 3D). Again, the total gene TPM significantly outperformed the median gene TPM (Wilcoxon Test, *p* < .05, Figure 3E). The total gene TPM was also slightly higher than the maximum TPM in these datasets; the difference was, however, not significant. Also, the values of the three metrics were distributed in a similar way (Kruskal-Wallis test, *p* = .4637). Scaling the TPM values considering the number of proteins in which the peptide is found did again not improve performance **(**Wilcoxon Test, *p* > .05 Figure 3F).

It is also possible that a peptide occurs multiple times in a single protein sequence, e.g., in the case of repeating amino acid sequences (Luo and Nijveen, 2014). In our calculations above, such peptides were only counted once and we did not see an increase in performance when we considered duplicate peptides in a protein (Supplementary Figure S3). This is likely due to the fact that 99.6% of the peptides in the human proteome do not occur multiple times in the same protein.

Taken together, we used pepX successfully to retrieve abundance levels of peptides’ source proteins and showed that in cases where peptides can be retrieved from multiple proteins, summing up the TPM values of the encoding genes performs best in distinguishing ligands from decoy peptides.

### Transcript-level TPM data provides a more accurate estimation of peptide abundance than gene-level TPM data

**FIGURE 4:**
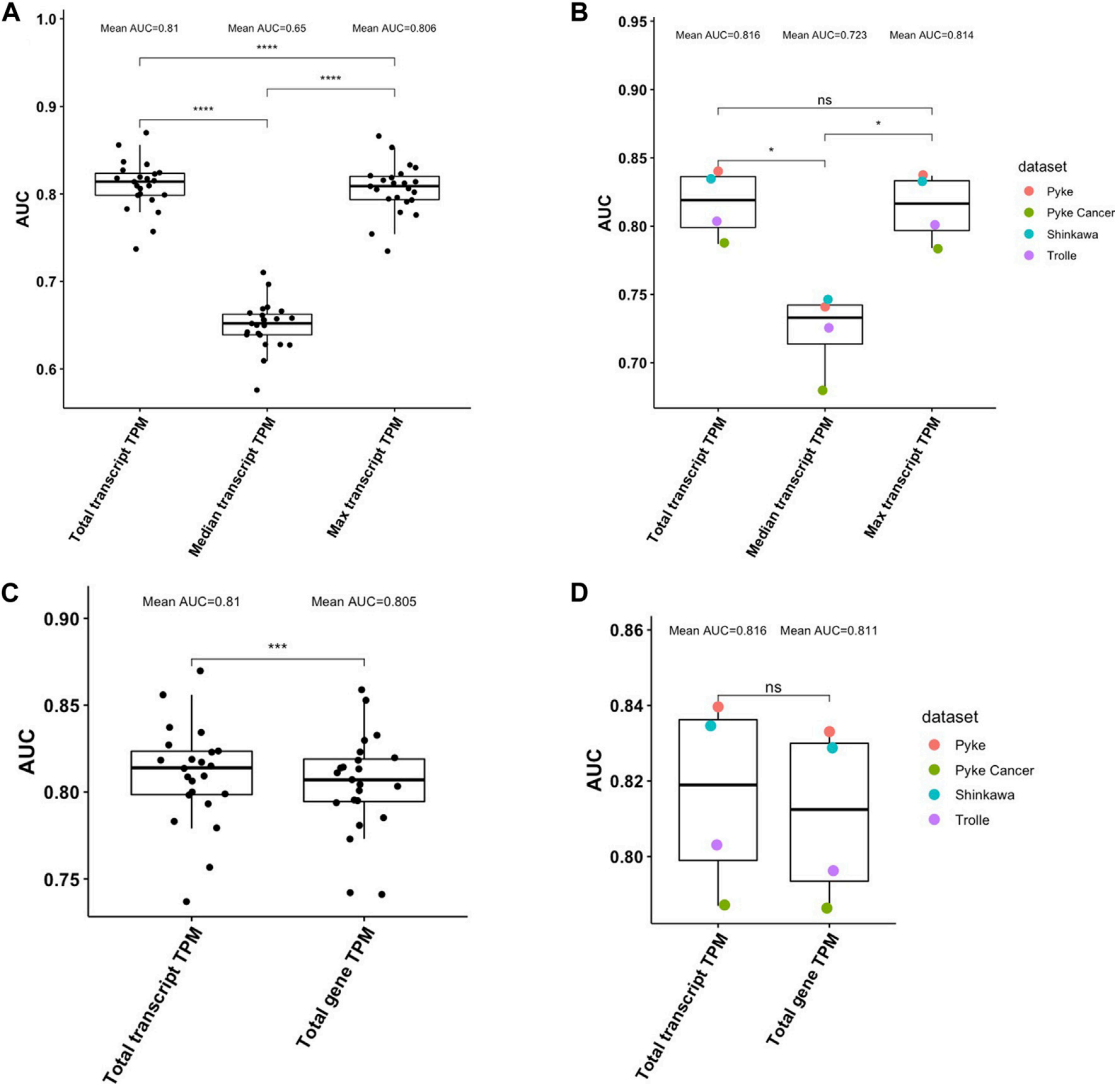
**(A)**. Performance comparison of different ways to aggregate transcript-level TPM values of multiple source proteins in distinguishing ligands of the HLA Ligand Atlas from decoy peptides. Summing up TPM values (total TPM) from all transcripts a peptide can be retrieved from performs best, followed by using the maximum TPM of all transcripts (Wilcoxon Test, p≤.0001). **(B)**. Performance comparison of different ways to aggregate transcript-level TPM values of multiple source proteins in distinguishing ligands of the four validation datasets from decoy peptides. **(C)**. Performance comparison of using transcript-level and gene-level TPM values in distinguishing ligands of the HLA Ligand Atlas from decoy peptides. The total transcript-level TPM significantly outperformed the total gene-level TPM (Wilcoxon Test, p≤.0001). **(D)**. Performance comparison of using transcript-level and gene-level TPM values in distinguishing ligands of the four validation datasets from decoy peptides. The mean AUC of the total transcript-level TPM is higher the total gene-level TPM, however not significantly (Wilcoxon Test, *p* > .05).

We next investigated how well peptide abundance can be estimated using transcript-level instead of gene-level TPM values. We again used pepX to retrieve transcript-level TPM values from HPA for the peptides from the HLA Ligand Atlas and the set of random decoy peptides. We evaluated the performance of using 1) the median TPM of all transcripts, 2) using the maximum TPM among all transcripts, and 3) summing up TPM values of all transcripts. Similar to what we have observed when using gene-level TPM, summing up the TPM values of all transcripts (total TPM) significantly outperformed the median and the maximum TPM in the HLA Ligand Atlas dataset (Kruskal-Wallis test, *p* < .0001, Wilcoxon Test, p≤.0001, Figure 4A). In the validation datasets, the total TPM also significantly outperformed the median TPM (Kruskal-Wallis test, *p* < .05, Wilcoxon Test, *p* < .05, Figure 4B). With a mean AUC of .816 the total TPM performed slightly better than the maximum TPM with a mean AUC .814; the difference was, however, not significant (Wilcoxon Test, *p* > .05).

Comparing the total gene TPM and the total transcript TPM for the peptides from HLA Ligand Atlas and the set of random decoy peptides showed that transcript-level TPM values perform significantly better than gene-level TPM values **(**Wilcoxon Test, p≤.001, Figure 4C). On the validation datasets, with a mean AUC of .816, transcript-level total TPM performed better than the gene-level total TPM with an AUC of .811, however not significantly (Wilcoxon Test, *p* > .05, Figure 4D).

The Genotype-Tissue Expression Project (GTEx) is another database that provides tissue-specific gene expression data from healthy tissue samples. We wanted to compare the performance of using GTEx and HPA transcript-level expression data for estimating the abundance of the peptides from the HLA Ligand Atlas. We focused on the 18 tissue types that we could clearly match between the three datasets **(** Supplementary Table S1). For this subset of peptides, using TPM values from HPA significantly outperformed using TPM values from GTEx (AUC of .812 vs. .805, Wilcoxon Test *p* < .01, **S** Supplementary Figure S4).

All expression databases provide transcript-level TPM values calculated with RSEM (Li and Dewey, 2011). The TCGA also provided TPM values calculated with Kallisto (Bray et al., 2016). We compared the performance of the two metrics using the Pyke Cancer dataset. The two metrics performed very similarly: the TPM calculated using RSEM had an AUC of .787 and the one calculated using Kallisto had an AUC of .786.

Taken together, we have shown here that, if available, transcript-level TPM data should be used to estimate peptide abundance, regardless if RSEM or Kallisto was used to calculate the TPM values. In the case of expression data of healthy tissue, HPA seems to be slightly more accurate for estimating peptide abundance of ligands eluted from healthy tissue samples.

## Discussion

The abundance of a peptide’s source antigen can play an important factor in predicting the likelihood that the peptide is a ligand and an epitope that is recognized by T cells, as it has previously been demonstrated that high peptide abundance can compensate for poor binding affinity (Kosaloglu-Yalcin et al., 2022). Although several novel prediction tools now integrate the expression levels of source antigens, there were, to our knowledge, no web tools available that can help users in retrieving such data from numerous public databases. We developed pepX to fill this gap. We also formally analyzed the different ways expression data can be retrieved and aggregated to most accurately estimate peptide abundance. We successfully used pepX to estimate peptide abundance for ligands from several datasets of eluted ligands and showed that summing up the transcript-level TPM values of different possible source proteins provides the most accurate estimation.

Of the 130,949 peptides of length 8–14 in the HLA Ligand Atlas, 5,332 peptides could not be matched using pepX. This might be due to differences between the reference proteome sources, versions, and filters applied in each project. For instance, the HLA Ligand Atlas relied on the SwissProt human reference proteome while pepX makes use of the Ensembl reference. We are currently exploring ways to further expand the Universe of peptides that can be quantified using pepX. To enable the quantification of MHC class II presented peptides, we have included 15mer peptides in pepX and are planning to include peptide lengths of up to 25 in the next version of pepX.

pepX currently outputs Ensembl identifiers (gene, transcript, and protein ids) and HGNC gene symbols. These identifiers can be mapped with an external id mapping tool, such as provided by UniProt (UniProt Consortium, 2019) or Biomart (Smedley et al., 2009). We included six gene expression datasets in pepX, namely HPA, GTEx, TCGA, CCLE, and RNA-Seq data of a B721.221 cell line. We anticipate adding more datasets, including expression values from mouse samples. We also plan to provide the option to upload custom TPM tables, e.g., from patient RNA-Seq, which can be used to retrieve peptide abundance estimates.

Including peptide abundance was shown to improve accuracy when predicting naturally eluted ligands, cancer epitopes, and epitopes from infectious diseases such as SARS-CoV-2 (Sarkizova et al., 2020; Garcia Alvarez et al., 2022; Kosaloglu-Yalcin et al., 2022). pepX can be used in combination with epitope prediction tools, that consider peptide abundance, such as HLAthena (Sarkizova et al., 2020), AXEL-F (7), and NetMHCpanExp (Garcia Alvarez et al., 2022): the user would first use pepX to retrieve peptide abundance values and use those results as an input for their preferred epitope prediction tool. In a future release of the IEDB Analysis Resource, we also plan to add the option to pipe pepX results directly to epitope prediction tools. We are also working on allowing users to upload custom TPM values to be used when annotating the uploaded peptides.

As pepX is built from the human reference proteome, it is currently not possible to search for mutated peptides, e.g., neoantigens. In a future version of pepX we are planning to provide the option to search for mutated peptides as well by incorporating an initial scan with PepMatch (manuscript under review). PepMatch is a sequence comparison tool we developed that searches a given proteome for exact peptide matches, matches with a defined tolerance for mismatching residues, and best matches. PEPMatch uses a k-mer mapping algorithm, which preprocesses proteomes prior to searching, and achieves a 50-fold increase in speed over algorithms, such as BLAST, while also guaranteeing accurate results. Combined with the option to upload patient-specific RNA-Seq data, this will make pepX highly valuable in selecting and prioritizing neoantigens for immunotherapeutic approaches.

pepX is freely available at http://tools.iedb.org/pepx and will be periodically updated to include additional features that provide more utility.

## Data Availability

The datasets presented in this study can be found in online repositories. The names of the repository/repositories and accession number(s) can be found in the article/Supplementary Material.
