# Supplementary material for: Estimating tissue-specific peptide abundance from public RNA-Seq data
Source: Front Genet. 2023 Jan 12;14:1082168. doi: 10.3389/fgene.2023.1082168 (PMC9878344; doi:10.3389/fgene.2023.1082168)
Supplement: Supplementary file 1 [file DataSheet1.PDF]

Supplemental Data

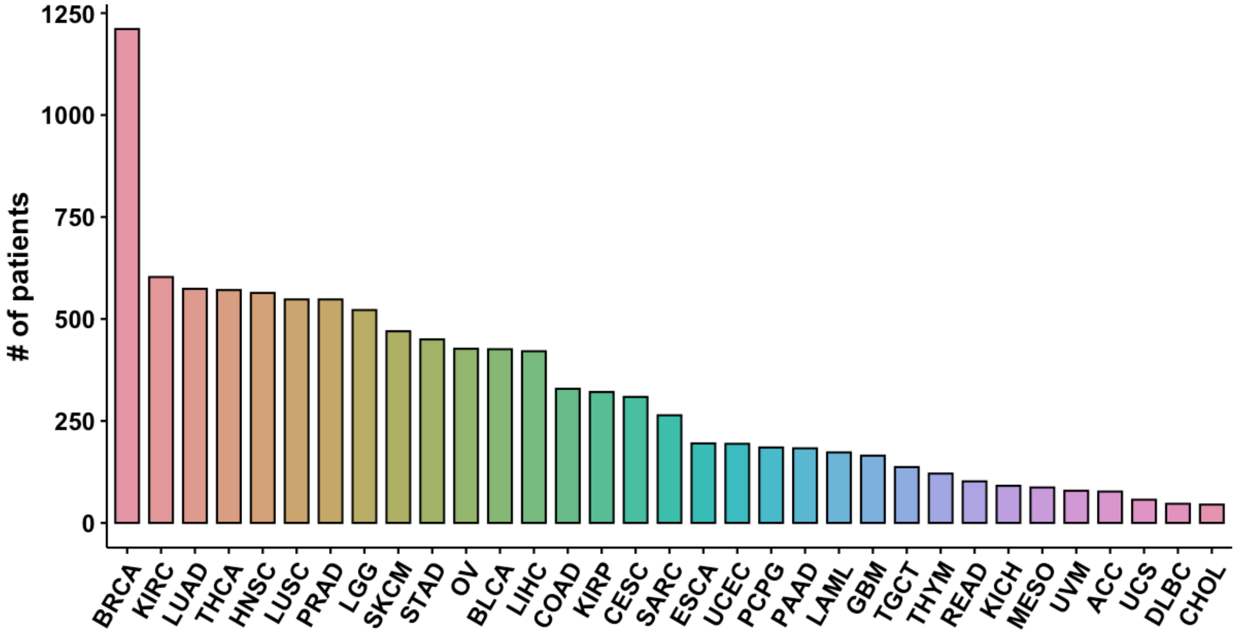

Supplemental Figure S1
The number of samples for each tumor type that were sequenced as part of the TCGA.

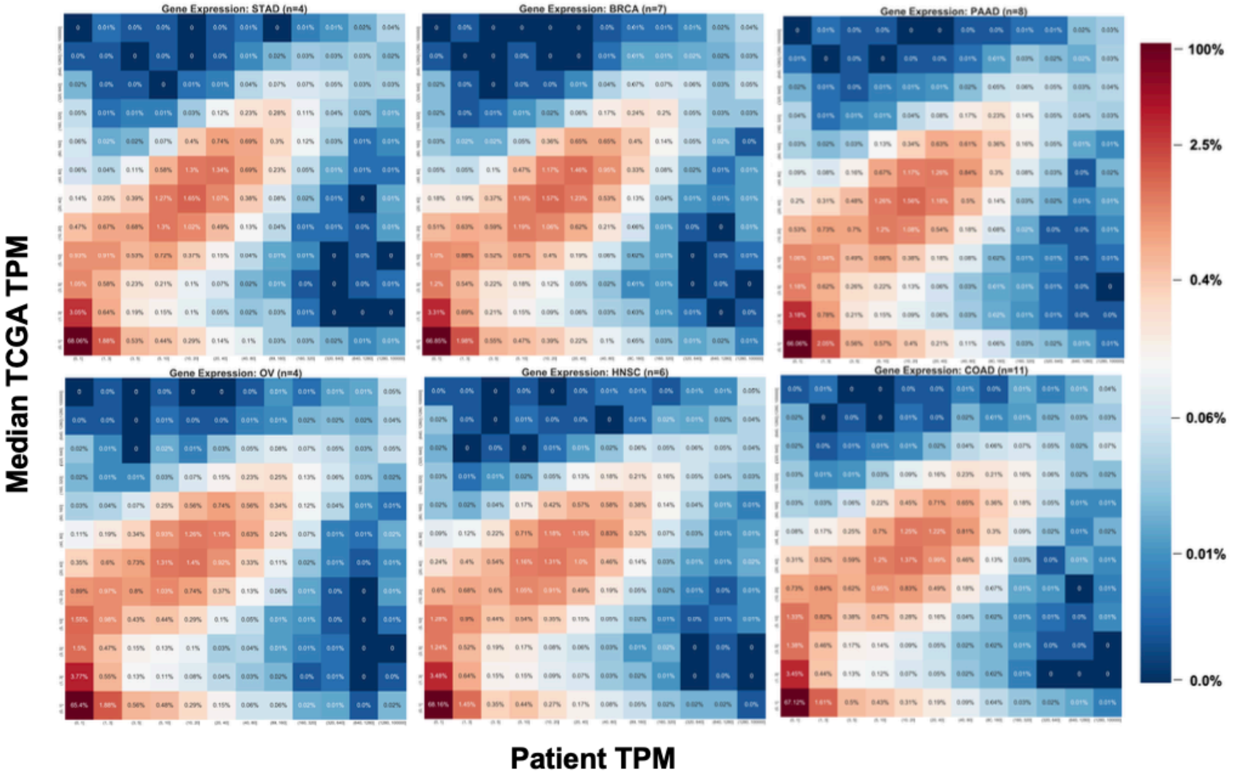

Supplemental Figure S2

Correlation between TPM values derived from patient-specific RNA-Seq and TCGA for six different histologies: stomach adenocarcinoma (STAD), breast carcinoma (BRCA), pancreatic adenocarcinoma (PAAD), ovarian carcinoma (OV), head and neck squamous cell carcinoma (HNSC), colon adenocarcinoma (COAD). For each patient, the TPM values were separated into ranges for both the patient-specific (x-axis) and the TCGA median (y-axis) TPM values. For each TPM range combination, the fraction of genes expressed within the corresponding TPM ranges is shown as a percentage and is also color-coded.

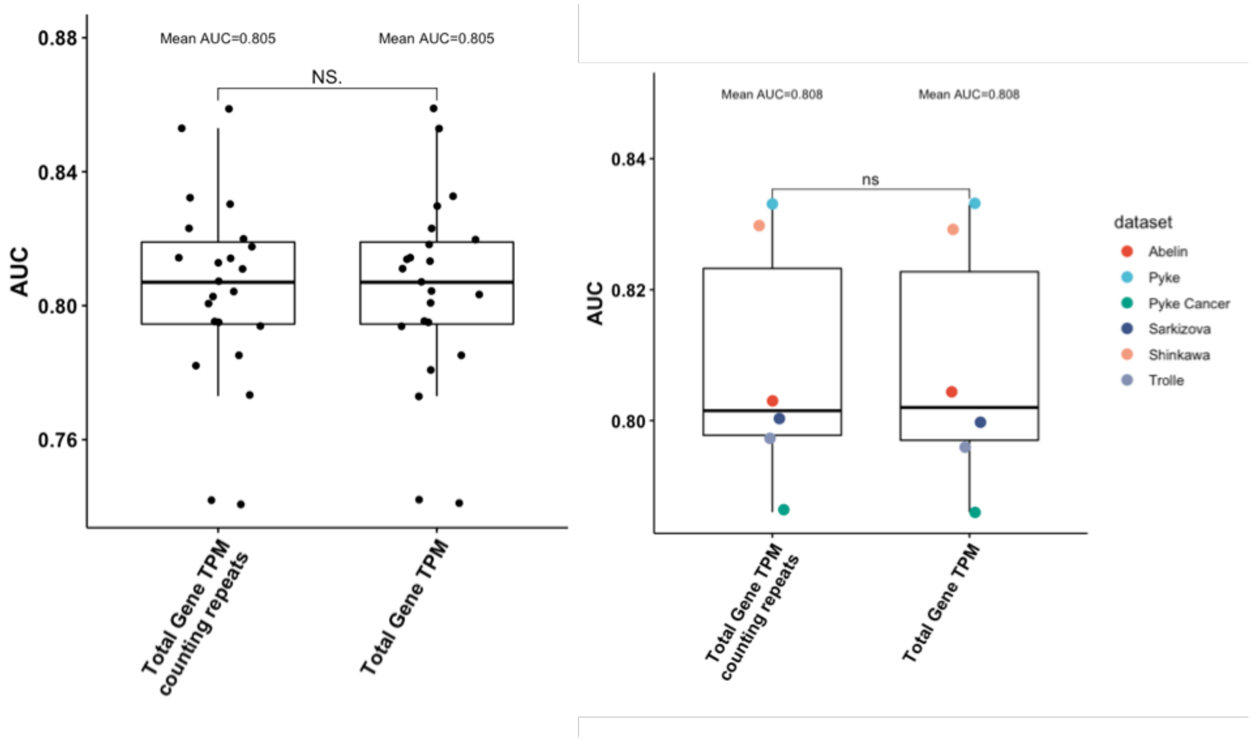

Supplemental Figure S3

Performance of pepX when considering duplicate peptides in a protein vs. not in the HLA Ligand dataset and the validation datasets.

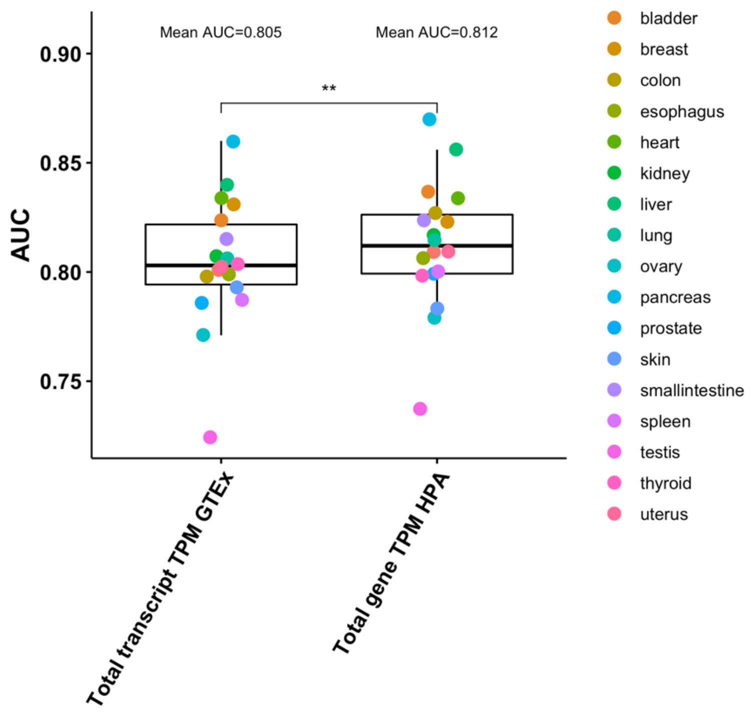

Supplemental Figure S4
pepX performance on predicting peptides from the HLA Ligand Atlas when using GTEX expression data vs HPA.

Supplemental Table S1

HLA Ligand Atlas	HPA Subtype	GTEX Subtype
Adrenal_gland	adrenal gland	Adrenal Gland
Brain	-	Brain
Mamma	breast	Breast
Colon	colon	Colon
Uterus	endometrium	Uterus
Esophagus	esophagus	Esophagus
Heart	heart muscle	Heart
Kidney	kidney	Kidney
Liver	liver	Lung
Lung	lung	Liver
Muscle	-	Muscle
Ovary	ovary	Ovary
Pancreas	pancreas	Pancreas
Prostate	prostate	Prostate
Skin	skin	Skin
**Small_intestine**	small intestine	Small Intestine

**Spleen**	spleen	Spleen
**Testis**	testis	Testis
**Thyroid**	thyroid gland	Thyroid
**Bladder**	urinary bladder	Bladder
